# A pluripotent stem cell atlas of multilineage differentiation

**DOI:** 10.1038/s41597-025-05549-w

**Published:** 2025-07-15

**Authors:** Sophie Shen, Tessa Werner, Han Sheng Chiu, Xiaoli Chen, Quan Nguyen, Nathan J. Palpant

**Affiliations:** Institute for Molecular Bioscience, The University of Queensland, St Lucia, QLD 4067 Australia

**Keywords:** Stem-cell differentiation, Gene expression

## Abstract

Human pluripotent stem cells offer a scalable platform to study genetic and signalling mechanisms governing cell lineage decisions during differentiation. Genome-wide and single-cell transcriptomics technologies likewise offer high-throughput analysis of heterogeneous cell differentiation states. While *in vivo* development has been extensively characterised using these technologies, there remains a need for comprehensive single-cell transcriptomic profiling of stem cell differentiation from pluripotency. Understanding gene expression changes governing differentiation *in vitro* is key to developing high fidelity differentiation protocols and understanding fundamental mechanisms of development. We generated a single-cell RNA sequencing time course to study the role of developmental signalling pathways on multilineage diversification from pluripotency *in vitro*. The combined dataset of over 60,000 cells spans cell types from a time course of differentiation across all germ layers, ranging from gastrulation cell states to progenitor and committed cell types. These data provide a diverse benchmarking reference point to compare against *in vivo* development and advance understanding of signalling regulation of differentiation, providing insights into protocol development, drug screening, and regenerative medicine applications.

## Background

Cell differentiation is governed by sequential signalling cues that coordinate gene expression changes to guide specification of functionally distinct cell types. Human pluripotent stem cells (hPSCs) represent a controlled and scalable platform to study this process in a human system, providing models and products with broad potential applicability across drug screening, disease modelling, and cell therapy^1,2^. Numerous differentiation protocols have been designed to derive diverse cell types from pluripotency through modulation of molecular, temporal, and structural parameters of culture conditions to achieve differentiated cell phenotypes in a dish^3^. There, however, is little coordination in the field for controlled benchmarking of each parameter and its effects on differentiation of cell lineages^4^, limiting reproducibility and optimisation of protocols for therapeutic use. We aim to address this by providing a study that evaluates effects of a restricted set of signalling cues at the germ layer stage of differentiation, while controlling for all other variables. We harness the opportunity provided by high-throughput transcriptomics to characterise mesendoderm-directed multilineage differentiation and provide an *in vitro* reference point to benchmark against the many existing *in vivo* single-cell transcriptomic atlases of development^5,6^. This dataset serves as a resource to better understand early human lineage trajectories and to inform the design of robust and scalable differentiation protocols relevant to applications for regenerative medicine.

**Fig. 1:**
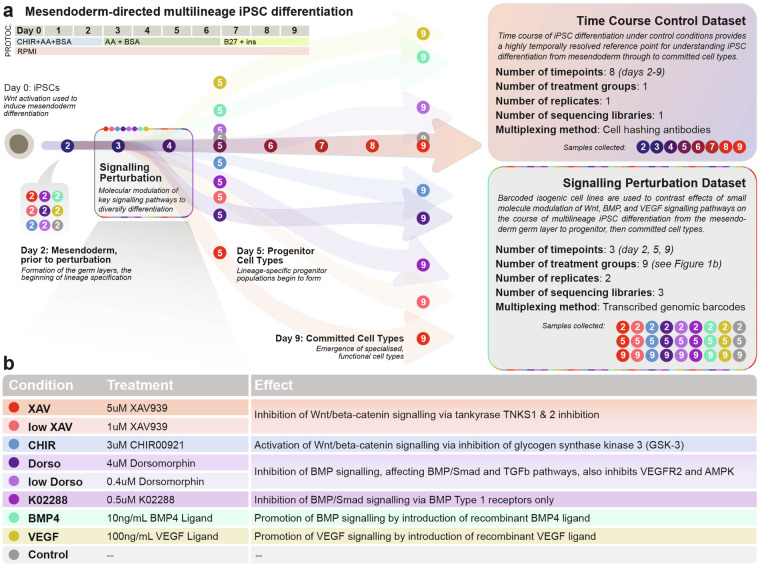
Experimental design and rationale. (**a**) High resolution time course dataset and small molecule signalling perturbation datasets were designed to complement each other as reference points for multilineage differentiation from pluripotency *in vitro*. Base differentiation protocol used in the time course dataset and added to for the signalling perturbation dataset is shown on the top left. Protoc.: Protocol; CHIR: CHIR00921; AA: Ascorbic Acid; ins: insulin. See **Methods** for more protocol details and Table 1 for details about time point/replicate/sequencing library assignment for each sample in the sequencing perturbation dataset. (**b**) Description of the molecules used in the signalling perturbation dataset.

We provide a two-part dataset. The first captures temporal regulation of gene expression changes during differentiation of human induced pluripotent stem cells (hiPSCs) into mesendoderm cell types over eight consecutive days of differentiation, spanning mesendodermal cells at day 2 to committed cell types at day 9 of differentiation (Fig. 1a). The second dataset interrogates the role of WNT, BMP4, and VEGF signalling pathways during differentiation through introduction of small molecule or recombinant proteins targeting these pathways at the germ layer stage of differentiation. The dataset captures the cells prior to treatment (day 2), and the resulting progenitor (day 5) and committed cell types (day 9) following these signalling perturbations (Fig. 1b & Table 1). The data are provided as two separate scRNA-seq datasets, as well as an integrated dataset with cell type annotation and characterisation. Our companion manuscript^7^ demonstrates utility for this integrated *in vitro* dataset to reveal a novel role for WNT signalling regulator *TMEM88* in cardiovascular development *in vivo*. In the same manner, this dataset can be further interrogated to uncover signalling-, temporal-, and lineage-specific regulators of differentiation. Overall, this single-cell transcriptomic atlas of hiPSC differentiation will be of interest to the field by providing a starting point for discovery and benchmarking against *in vivo* development to facilitate protocol development with relevance ranging from fundamental understanding of cell lineage regulation to applications in cell therapy and synthetic biology.

**Table 1:** Sample assignment in signalling perturbation dataset.

BC Line	Condition	Library 1	Library 2	Library 3	Concentration	Treatment	Source
BC01	Control	Day 5	Day 2	Day 9	—	—	—
BC02	Low XAV	Day 5	Day 2	Day 9	1uM	XAV939	STEMCELL Technologies #72674
BC03	XAV	Day 5	—	Day 9	5uM	XAV939	STEMCELL Technologies #72674
BC04	CHIR	Day 5	Day 2	Day 9	3uM	CHIR99021	STEMCELL Technologies #72054
BC05	Low Dorso	Day 5	Day 2	Day 9	0.4uM	Dorsomorphin	Sigma-Aldrich #P5499
BC06	Dorso	Day 5	Day 2	Day 9	4uM	Dorsomorphin	Sigma-Aldrich #P5499
BC07	K02288	Day 5	Day 2	Day 9	0.5uM	K02288	Cayman Chemical #16678
BC08	BMP4	Day 5	Day 2	Day 9	10 ng/mL	Recombinant Human BMP4	R&D Systems #RDS314BP010
BC11	VEGF	Day 5	Day 2	Day 9	100 ng/mL	Recombinant Human VEGF	R&D Systems #RDSPRD29350
BC10	Control	Day 2	Day 9	Day 5	—	—	—
BC09	Low XAV	Day 2	Day 9	Day 5	1uM	XAV939	STEMCELL Technologies #72674
BC12	XAV	Day 2	Day 9	Day 5	5uM	XAV939	STEMCELL Technologies #72674
BC13	CHIR	Day 2	Day 9	Day 5	3uM	CHIR99021	STEMCELL Technologies #72054
BC14	Low Dorso	Day 2	Day 9	Day 5	0.4uM	Dorsomorphin	Sigma-Aldrich #P5499
BC15	Dorso	Day 2	Day 9	Day 5	4uM	Dorsomorphin	Sigma-Aldrich #P5499
BC16	K02288	Day 2	Day 9	Day 5	0.5uM	K02288	Cayman Chemical #16678
BC17	BMP4	Day 2	Day 9	Day 5	10 ng/mL	Recombinant Human BMP4	R&D Systems #RDS314BP010
BC18	VEGF	Day 2	Day 9	Day 5	100 ng/mL	Recombinant Human VEGF	R&D Systems #RDSPRD29350

Note: BC stands for genomic barcode. The BC03 line in Library 2 was not sequenced.

## Methods

### Cell lines and maintenance

All human pluripotent stem cell studies were carried out in accordance with consent from the University of Queensland’s Institutional Human Research Ethics approval (HREC# 2015001434). Undifferentiated hiPSCs were cultured on Vitronectin XF (STEMCELL Technologies #07180)-coated plates in mTeSR1 media (STEMCELL Technologies #05850) with supplement at 37 °C with 5% CO2.

The cell line used to generate the time course dataset was a WTC CRISPRi TMEM88-g2.3 GCaMP hiPSC line (Karyotype: 46, XY; RRID: CVCL_VM38; generously provided by M. Mandegar and B. Conklin, Gladstone Institute, UCSF), generated as previously described^8^. In brief, a doxycycline-inducible pQM-u6g-CNKD construct containing guide RNA targeting the *TMEM88* transcriptional start site was transfected into WTC CRISPRi GCaMP hiPSCs using the GeneJuice protocol (Novagen). Cryopreserved WTC CRISPRi TMEM88-g2.3 GCaMP hiPSC samples were tested for copy number variants using the STEMCELL Technologies hPSC Genetic Analysis Kit (STEMCELL Technologies #07550), carried out by StemCore (Brisbane, Australia). For this study, the cells were not exposed to doxycycline and were treated as transcriptionally wildtype. For the signalling perturbation dataset, eighteen custom barcoded hiPSC lines were used. To generate the barcoding lines, WTC-WT11 hiPSCs (Gladstone Institute of Cardiovascular Research, UCSF; Karyotype: 46, XY; RRID: CVCL_Y803, generated as previously described^9,10^) were edited to enable expression of a barcoded GFP transcript driven by a CAG promoter at the human *AAVS1* safe-harbour locus via CRISPR-Cas9 genome editing. These barcoding lines thus facilitate simple multiplexing of isogenic hiPSC scRNA-seq samples. 15 cells per barcoding line were karyotyped as a professional service by Sullivan Nicolaides Pathology (see accompanying manuscript^7^ for more details and quality control of the genomic barcoding strategy).

### Mesendoderm-directed differentiation

On the day prior to differentiation (day −1), cells were dissociated using 0.5 mM EDTA solution and seeded onto separate coated plates in mTESR1 pluripotency media with ROCK Inhibitor (STEMCELL Technologies #72308) and cultured overnight. Once forming an ~80% confluent monolayer, differentiation was induced (day 0) by changing the culture media to RPMI (ThermoFisher, #11845119) containing 3 µM CHIR99021 (STEMCELL Technologies, #72054), 500 mg/mL BSA (Sigma #A9418), and 213 mg/mL ascorbic acid (Sigma #A8960) with a PBS wash. On days 3 and 5, media was changed with the same media cocktail excluding CHIR99021. On day 7 and every subsequent second day, cultures were fed with RPMI containing 1xB27 (ThermoFisher #17504001) supplement plus insulin. See also Fig. 1a.

### Single-cell RNA sequencing for control time course dataset

Mesendoderm-directed differentiation as described above was induced a separate plate for each collection time point (days 2–9 of differentiation; Fig. 1a), with one biological and technical replicate per time point. Cells were dissociated using 0.5 mM EDTA in 2.5% Trypsin (ThermoFisher, #15400054) and neutralised with 50% foetal bovine serum (GE Healthcare Life Sciences, #SH30084.03) in DMEM/F12 media (Sigma #11320033). 1e6 cells from each sample were labelled with a different TotalSeq-A cell hashing antibody (BioLegend antibodies A2051-8) as per the recommended protocol^11^ and sorted for viability on a BD Influx Cell Sorter (BD Biosciences) using propidium iodide. 5e5 live cells per time point were collected and pooled for Chromium Single Cell 3′ V3 (10x Genomics) reactions following the manufacturer’s protocol, targeting 2e4 cells. The fraction of droplets containing the Cell Hashing Oligonucleotide (HTO)-derived cDNA were captured using HTO additive primers and Illumina TruSeq DNA D7xx_s primers according to the cell hashing protocol. Gene expression libraries were sequenced on an Illumina NovaSeq 6000 and the cell hashing libraries were sequenced on an Illumina NextSeq 550Dx. The cell ranger software (10x Genomics, version 3.0.2) was used to demultiplex base calls into fastq files and then map the reads to GRCh38 and derive gene expression count matrices. CITE-seq-Count^12^ (version 4.2.1) was used to quantify reads mapped to Cell Hashing HTOs for sample demultiplexing.

### Single-cell RNA sequencing for signalling perturbation dataset

Mesendoderm-directed differentiation was performed as described above in separate plates for each time point, treatment group, and biological replicate (Fig. 1a). To allow for biological duplicates for each of the treatment-timepoint pair, 18 barcoded cell lines were used (see **Methods: Cell lines and Maintenance** and Table 1). On day 2 of differentiation, one sample per cell line was collected for scRNA-seq to serve as an untreated reference point. For the remaining samples, cells were treated with a small molecule or recombinant protein signalling perturbation as detailed in Table 1, added to the existing media cocktail introduced on day 3 of differentiation and removed on day 5. Resulting cells were collected on day 5 and 9 of differentiation for scRNA-seq.

For cell collection, cells were dissociated using 0.5 mM EDTA in 2.5% Trypsin (ThermoFisher, #15400054) and neutralised with 50% foetal bovine serum (GE Healthcare Life Sciences, #SH30084.03) in RPMI media (ThermoFisher, #11845119). 5e5 cells per group were pooled into three sample pools (as per Table 1, where each “Library” corresponds to a sample pool) and sorted for viability on a BD Influx Cell Sorter (BD Biosciences) using propidium iodide, compensating for the GFP signal. Chromium Single Cell 3′ v3 (10x Genomics) reactions were performed for each sample pool according to the manufacturer’s protocol, targeting 2e4 cells. Gene expression libraries were prepared according to the manufacturer’s protocol and an additional aliquot of amplified full-length cDNA was used to generate a barcoding library for each sample pool, amplifying the cDNA regions containing the barcoding cassette and appending partial P5 and P7 sequencing adaptors. A single pool was prepared from the three gene expression and three barcoding libraries for sequencing. The samples were pooled equimolar within each library type and combined so that the gene expression libraries together made up 90% of the pool, and the barcoding libraries 10%. Samples were sequenced on an Illumina NovaSeq 6000 using a NovaSeq 6000 S4 Kit V1 (200 cycles) (Illumina, #20027466). Base cell demultiplexing, fastq file generation, and read mapping to GRCh38 to derive gene expression and barcode count matrices was achieved using the cell ranger pipeline (10x Genomics, version 3.1.0).

### Sample demultiplexing, cell filtering and data pre-preprocessing

**Fig. 2:**
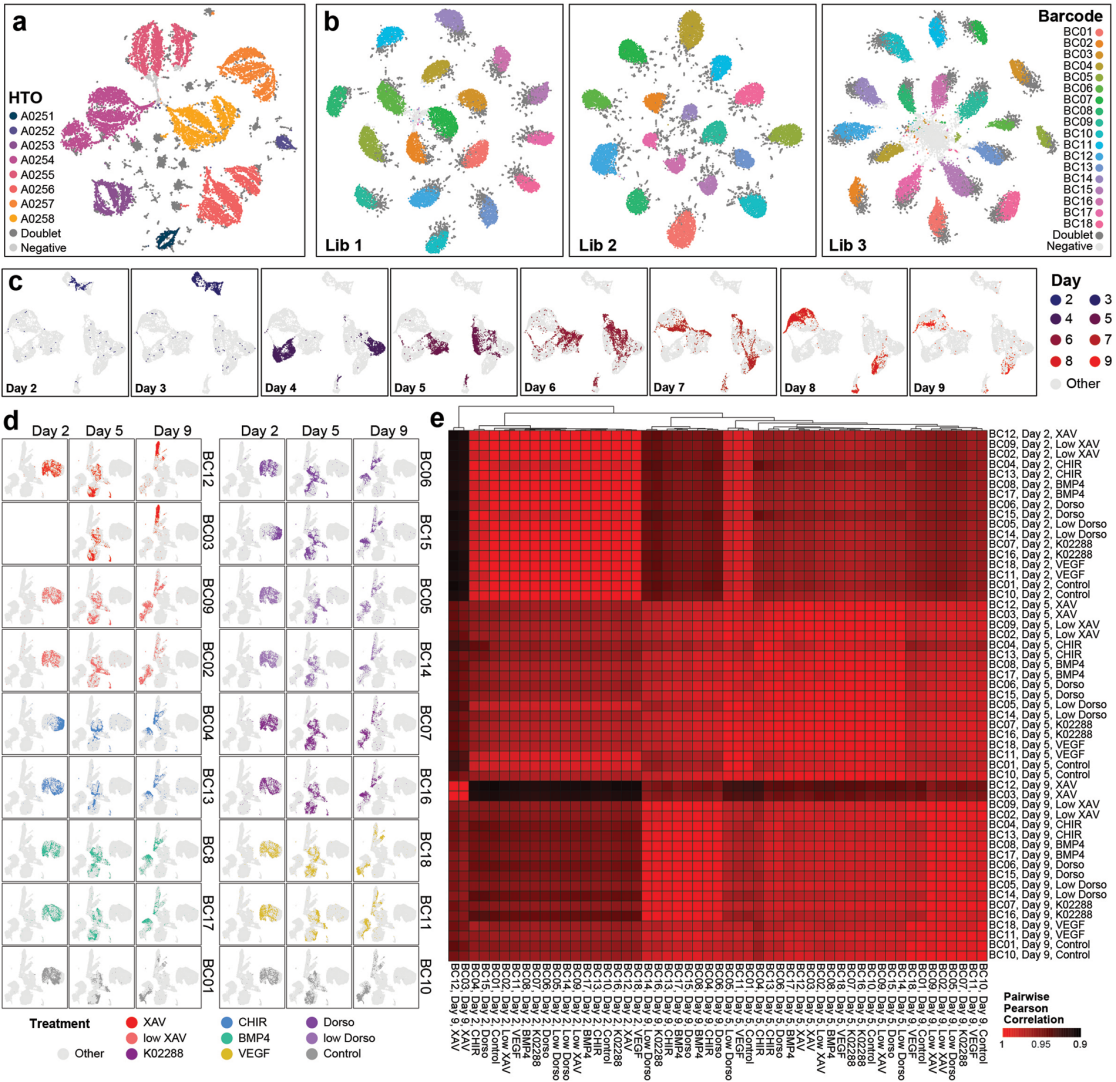
Cell hashing and genomic barcoding both facilitate effective sample multiplexing. (**a,b**) tSNE plots showing distribution of cells based on HTO (Cell Hashing Oligonucleotide; time course dataset; left) and genomic barcode reads (signalling perturbation dataset; right). (**c,d**) UMAPs showing distribution of cells in each timepoint, treatment, and barcoding line after quality control filtering (see Table 3) in the time course (**c**) and signalling perturbation (**d**) datasets. Further details about assignment of treatment groups to each sequencing library, timepoint, and barcode are shown in Table 1. (**e**) Pearson correlation between average gene expression of cells in each individual sample in the signalling perturbation dataset.

Sample demultiplexing, filtering, and pre-processing was performed on the two datasets separately. The *HTODemux* function in the *Seurat* R package^13^ was performed on the HTO and barcode count matrices for their respective datasets, using the default 0.99 quantile cutoff to determine the dominant sample barcode for each cell and identify negative and doublet cells based on their sample barcode counts. All cells annotated as “negative” based on the *HTODemux* output were removed as their sample of origin cannot be retrieved. We also used the three *in silico* doublet detection methods in the *scds* R package^14^ to identify cell barcodes likely to be doublets based on their transcriptomic features, so that doublets that share the same sample barcode could also be identified. Cells labelled as doublets by three or more methods (including sample barcoding) were removed. Finally, cell filtering based on the raw expression matrices was performed to retain only high quality cells with library sizes between 10,000 and 100,000 reads, 2,500 and 10,000 features, fewer than 20% of reads mapping to mitochondrial genes, and fewer than 45% to ribosomal genes for the time course dataset. For the signalling perturbation dataset, retained cells had with library sizes between 5,000 and 50,000 reads, 2,000 and 7,500 features, and mitochondrial reads making up fewer than 25% of all reads in each cell. Data normalisation (*NormalizeData*), scaling (*ScaleData*), PCA (*RunPCA*), and UMAP (*RunUMAP*) dimensionality reduction as part of the default *Seurat* pipeline were performed on each dataset separately for preliminary visualisation (Fig. 2c,d). For evaluation of replicate similarity (Fig. 2e), we averaged the gene expression of cells from each individual sample (barcoding line/time point/treatment combination, Table 1) and calculated the Pearson correlations between all sample pairs, visualising the results using the *pheatmap* R package. See also scripts *1_preprocess_timecourse.R*, *2_preprocess_signalling.R*, *3_FIG2.R*.

### Dataset integration and cell type annotation

Raw count matrices from the two scRNA-seq datasets were used as input for data integration using the RCPI method (*scMultiIntegrate*) as part of the *RISC* R package^15^, following the recommended pipeline for pre-processing. Genes expressed in both datasets were used to generate 15 gene eigenvectors using the signalling perturbation dataset as the reference dataset to perform integration, returning 50 principal components for further analysis. UMAP dimensionality reduction (*scUMAP*) of the integrated data was also done using the *RISC* package, using 15 components of the ‘PLS’ embeddings as recommended for the integrated values. Cell type clustering (*FindClusters* for resolutions 0.1, 0.2, 0.3 and 0.4) (Fig. 4a) was performed using the *Seurat* package using the first 10 PLS embeddings from the RCPI integration for the reduced dimension input, instead of the default PCA reductions. The *Seurat* function *FindAllMarkers* was used to find differentially expressed genes for each cluster at the 0.3 cluster resolution. For GO term and KEGG pathway enrichment analyses, the top 100 most significantly differentially expressed genes for each cluster (ranked by “p_val_adj” then “avg_log2FC”) were used as input for the *enrichGO* and *enrichKEGG* functions from the *clusterProfiler* R package^16^. For label transfer analysis to align our atlas dataset against *in vivo* development, we used the *Seurat* label transfer pipeline. First, both query and reference datasets underwent *TRIAGE-*transformation^17,18^ to prioritise the biological signal from cell type-specific regulatory genes before input into the *FindTransferAnchors* and *TransferData Seurat* functions with default parameters. The label transfer annotation for each cell were filtered by prediction score, selected based on the observed score distribution for each reference database separately (0.4 for the mouse organogenesis^19^ dataset, 0.3 for the human gastrulation^20^ dataset, and 0.05 for the prenatal mouse^6^ dataset). Cells that did not pass the score threshold are annotated as *NA* in the metadata and Fig. 4d. See also scripts *4_rcpi_integration.R*, *6_clustering_annotation.R, 7_label_transfer.R*.

## Data Records

All raw and processed data have been uploaded the NCBI Gene Expression Omnibus repository (accession ID: GSE279710^21^). The portion of the record described in this manuscript are labelled as “Signalling perturbations library” 1–3 (Samples GSM8578510-15), “Control time course” (Samples GSM8578516-17), and the supplementary (processed) files with the prefix “GSE279710_integrated_sig_time”. Raw mapped read data (fastq files) are provided in “GSE279710_RAW.tar”.

For the signalling perturbation dataset, there is one sample for the barcoding sequencing library (labelled as “BC”; e.g. GSM8578510 for library 1) and one sample for the gene expression sequencing data (labelled as “GEX”; e.g. GSM8578511 for library 1). Both the BC and GEX libraries are provided as three separate files: cell barcodes (e.g. GSM8578510_raw_barcode_sig1_barcodes.tsv.gz), gene (or feature) names (e.g. GSM8578510_raw_barcode_sig1_features.tsv.gz), and the gene expression count matrix (e.g. GSM8578510_raw_barcode_sig1_matrix.mtx.gz). Cell barcode files are a single column containing a list of all cell barcodes in the dataset. Feature files contain three columns: Ensembl ID, gene symbol, and library type (“Gene Expression”), where the BC files contain the barcode names in the first two columns, and the GEX files also have all 20 barcodes appended to the end of the features file, prefixed with “addSeq_”. The count matrix is a sparse matrix of raw read counts with columns as cell barcodes and rows as genes, in the same order as they appear in the corresponding barcode and feature files.

Note that for the signalling perturbation dataset, the barcode IDs were initially BC03-BC20 and renamed to be BC01-BC18 in the manuscript files. The initial numbering is reflected in the raw matrix files (Samples GSM8578510, 12 & 14), but not in the supplementary (processed) files in the record.

The raw files for the time course dataset are organised the same as the signalling perturbation dataset, where there is one sample for the sample multiplexing library (“Control time course, HTO”, sample GSM8578516), and one for the gene expression (“Control time course, GEX”, sample GSM8578517), each containing a cell barcode, feature, and count matrix file. In the time course GEX sample, the sample (HTO) barcodes are not included in the features and count matrix.

The supplementary files (i.e. the processed files with “GSE279710_integrated_sig_time” prefixes), also include a gene (GSE279710_integrated_sig_time_genes.txt.gz) and count matrix file (GSE279710_integrated_sig_time_norm.mtx.gz), where the count matrix is a sparse matrix containing the read counts after integration and normalisation as described in the **Methods**. Instead of a cell barcode file, a cell metadata file is provided (GSE279710_integrated_sig_time_meta.txt.gz) with the following columns providing extra information for each cell: “cell_barcode”; “UMAP1” & “UMAP2” (coordinates used in Fig. 4a); “run” (sequencing sample, where “0Xav” represents the time course dataset and “lib1”, “lib2”, and “lib3” represent the three libraries in the signalling perturbation dataset (see Table 1); “day” (time point); “treatment” (where “no_treatment_tc” represents the time course dataset, and details about the signalling perturbations are provided in Table 1); as well as several columns related to the *TRIAGE-Cluster* analysis introduced in our main manuscript^7,18,22^ (columns: “peak_cluster” (*TRIAGE-Cluster* cluster assignments), “PijuanSala_LabelTransfer” (filtered label transfer assignments with reference to a scRNA-seq dataset of mouse organogenesis^19^), “Hutchins_CTDomains” (filtered gene set scoring assignments using gene sets derived from RNA-seq of mouse embryonic domains^23^), “Tyser_GastrulaLT” (filtered label transfer assignments with reference to a scRNA-seq dataset of human gastrulation^20^), “Annotation” (cell type label as seen in our main manuscript^7^), and “Anchor Gene” (high scoring *TRIAGE* gene used to guide *TRIAGE-*clustering^22^)).

## Technical Validation

### Single-cell RNA sequencing and quality control

We generated the time course control and signalling perturbation scRNA-seq datasets separately, with the former captured in one Chromium Single Cell 3′ V3 (10x Genomics) reaction using Cell Hashing^11^ for sample multiplexing and minimisation of between-reaction batch effects. This dataset comprises eight time points separated by 24 hours from day 2 to 9 of *in vitro* mesendoderm differentiation, with one biological replicate per time point (eight samples, with one Cell Hashing antibody each) (Fig. 1a). We note that the cell line used for the time course experiment was found to have a 1.5-fold expression increase at the Chr20q11.21 locus compared to validated diploid controls, indicating a copy number expansion which could have an impact on phenotype (see **Methods**).

The signalling perturbation datasets captured cells across three Chromium reactions, profiling effects of perturbing differentiation with each of eight small molecule or recombinant proteins to modulate key developmental signalling pathways. Differentiating cells were captured at day 2 (mesendoderm cells prior to treatment), day 5 (progenitor cell types immediately following treatment), and day 9 (resulting definitive cell types), with two biological replicates, facilitated by use of eighteen isogenic barcoded cell lines for sample multiplexing to minimise costs and sequencing batch effects^7^ (Fig. 1a). Biological duplicates were collected and sequenced in different libraries to further allow for identification of between-library batch effects in data processing (Table 1). Comprehensive validation showing karyotypic and phenotypic normality of all isogenic barcoded cell lines is provided in our accompanying study^7^. After single-cell capture and RNA sequencing, the cell ranger pipeline (10x Genomics, see **Methods**) was used to generate fastq files and map reads to the GRCh38 genome to derive four gene expression matrices (one per reaction/sequencing library). Detailed read and alignment quality metrics are provided in Table 2. Standard scRNA-seq quality control metrics were assessed based on these gene expression matrices, demonstrating comparable metrics between the four libraries (Table 3).

**Table 2:** Sequencing quality control metrics.

Quality Control Metric	Time Course Library	Signalling Library 1	Signalling Library 2	Signalling Library 3
Estimated Number of Cells	20,000	24,721	24,828	26,583
Number of Reads	1,569,753,067	2,555,448,126	2,877,595,613	2,578,865,601
Mean Reads per Cell	78,487	103,372	115,901	97,012
% Q30 Bases in Barcode	96.8	96.7	96.6	96.7
% Q30 Bases in RNA read	95.5	95.2	94.9	95.3
% Q30 Bases in UMI	96.6	96.5	96.5	96.5
% Confident Mapping to Genome	94.5	93.7	93	94.1
% Confident Mapping to Transcriptome	60.5	58.2	57.7	58.1
% Fraction Reads in Cells	96.8	92.7	84.9	93

**Table 3:** Transcriptome quality control metrics and filtering.

Quality Control Metric	Time Course Library	Signalling Library 1	Signalling Library 2	Signalling Library 3
Pre-filter cell count	19,997	19,656	19,708	19,349
Median library size	23,575	22,812	21,801	21,063
Median number of genes	4,984	4,926	4,761	4,992
Median percentage of mitochondrial reads	5.83	6.89	8.06	5.86
Median percentage of ribosomal reads	27.93	29.15	27.29	21.63
Number of negative multiplexing barcodes*	349	426	25	2,016
Number of multiplexing barcode doublets	4,017	4,685	4,419	4,611
Number of *scds* doublets by 3 methods	1,306	893	1,032	500
Number of *scds* doublets by 2 methods	1,546	931	1,076	830
Number of *scds* doublets by 1 method	2,391	1,744	1,669	998
Number of doublets called by ≥ 2 methods (removed)	3,314	2,795	3,156	1,886
Final cell count after filtering (high quality singlets)	13,682	16,586	16,391	15,549

*Multiplexing barcodes refer to Cell Hashing HTOs for the time course library and genomic barcodes for the signalling libraries.

As both multiplexing methods relied on quantification of reads mapped to each sample barcode for sample demultiplexing (see also **Methods**), we performed tSNE dimensionality reduction of the cells based on their sample barcode expression (cell hashing oligonucleotide (HTO) barcodes for the time course dataset and genomic barcode for the signalling perturbation dataset) (Fig. 2a,b). This, alongside the *HTODemux* function in the *Seurat* package^13^ allowed identification of cells with multiplet or negative barcode reads (see accompanying study^7^, for more in-depth assessment of genomic barcoding quality), which were removed. *Scds*^14^, a transcriptome-based multiplet detection tool was also used to identify doublets via three different algorithms (Table 3). Combining the barcode-based and three transcriptome-based doublet detection methods, we removed cells predicted to be doublets by three or more methods, with the predicted doublets being assigned the barcode with the highest expression. Finally, cell filtering was performed to remove low quality, negative, and stressed cells based on their total read count, total gene count, mitochondrial, and ribosomal read content (Table 3 & **Methods**).

After filtering, the libraries had between 13,000 and 16,600 high quality singlets remaining (Table 3). We performed data scaling, normalisation, and dimensionality reduction on the time course and signalling perturbation datasets separately. UMAP representation of the time course dataset confirmed co-localisation of cells from the same time point (Fig. 2c). The same is true for the signalling perturbation dataset, where the UMAP visualisation and pairwise Pearson correlation between mean expression profiles of each sample indicate that biological replicates from the same time point (see also Table 1) but captured in different libraries have highly similar transcriptomic profiles that distinguish them from the other time points (Fig. 2d,e). While all samples at day 2 are treated identically, and largely form a single transcriptional cluster, we note that two samples (BC04 and BC15) appear to be more transcriptionally distinct, reflecting modest baseline variation between lines prior to treatment (Fig. 2d).

### Data integration

**Fig. 3:**
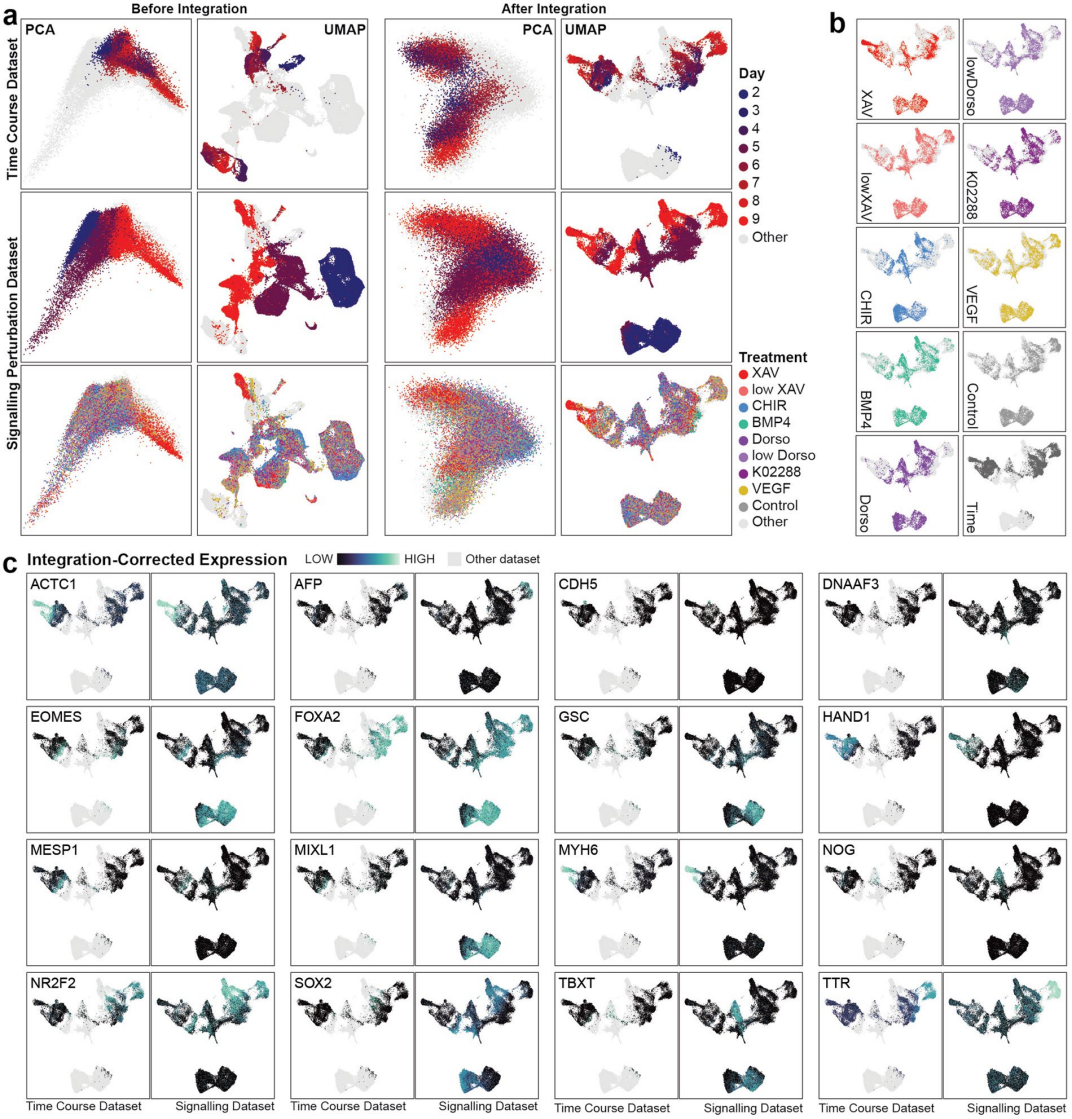
RCPI integration aligns similar cell types from each dataset to form the integrated data atlas. (**a**) PCA (left) and UMAP (right) representations of the datasets before (left) and after (right) RCPI data integration. Time course dataset (top row) is coloured by time point and signalling perturbation dataset (lower two rows) is coloured by both time point and treatment condition. (**b**) Distribution of cells from each treatment group in an integrated UMAP. (**c**) UMAPs showing alignment of select gene expression patterns between datasets of origin, where in each pair of plots cells from the time course dataset are shown on the left and cells from the signalling perturbation dataset on the right. For all plots, light grey “other” points indicate position of cells in the opposite dataset.

To address potential batch effects and improve comparability between the two datasets, we performed data integration using the RCPI algorithm^15^. Figure 3a shows principal components analysis (PCA) and UMAP representations of the combined datasets before integration, where the two datasets are almost entirely non-overlapping and transcriptionally distinct. After data integration, cells from the shared day 2, 5, and 9 time points co-localise. As anticipated, the cell types in the time course dataset are represented in the signalling perturbation dataset, with additional new cell types being generated in the latter as a result of the small molecule and recombinant protein treatments (Fig. 3a,b).

We visualise marker gene expression compared between the two datasets in the integrated UMAP space to further validate the integration and to gain insight into cell types captured in the dataset (Fig. 3c). This shows that mesoderm (*MESP1*, *EOMES*), definitive endoderm (*FOXA2*), endothelium (*CDH5*), posterior foregut (*TTR*), and cardiomyocytes (*ACTC1*, *MYH6*) are shared cell types between the two datasets, cell types all expected to arise from a largely undirected mesendoderm differentiation^3,24^. This marker gene expression analysis also highlights divergence of the signalling perturbation dataset from the time course to produce unique populations of cells expressing *NR2F2*, *SOX2*, *TBXT*, *NOG*, and *DNAAF3*. Finally, Fig. 3c also shows that the increased sampling of cells at day 2 in the signalling perturbation dataset reveals two transcriptionally distinct cell types at day 2, one enriched in mesendodermal marker *GSC*, and the other in *SOX2*, a gene associated with pluripotency and neuromesodermal lineages^25^.

### Clustering and cell type annotation in the integrated dataset

**Fig. 4:**
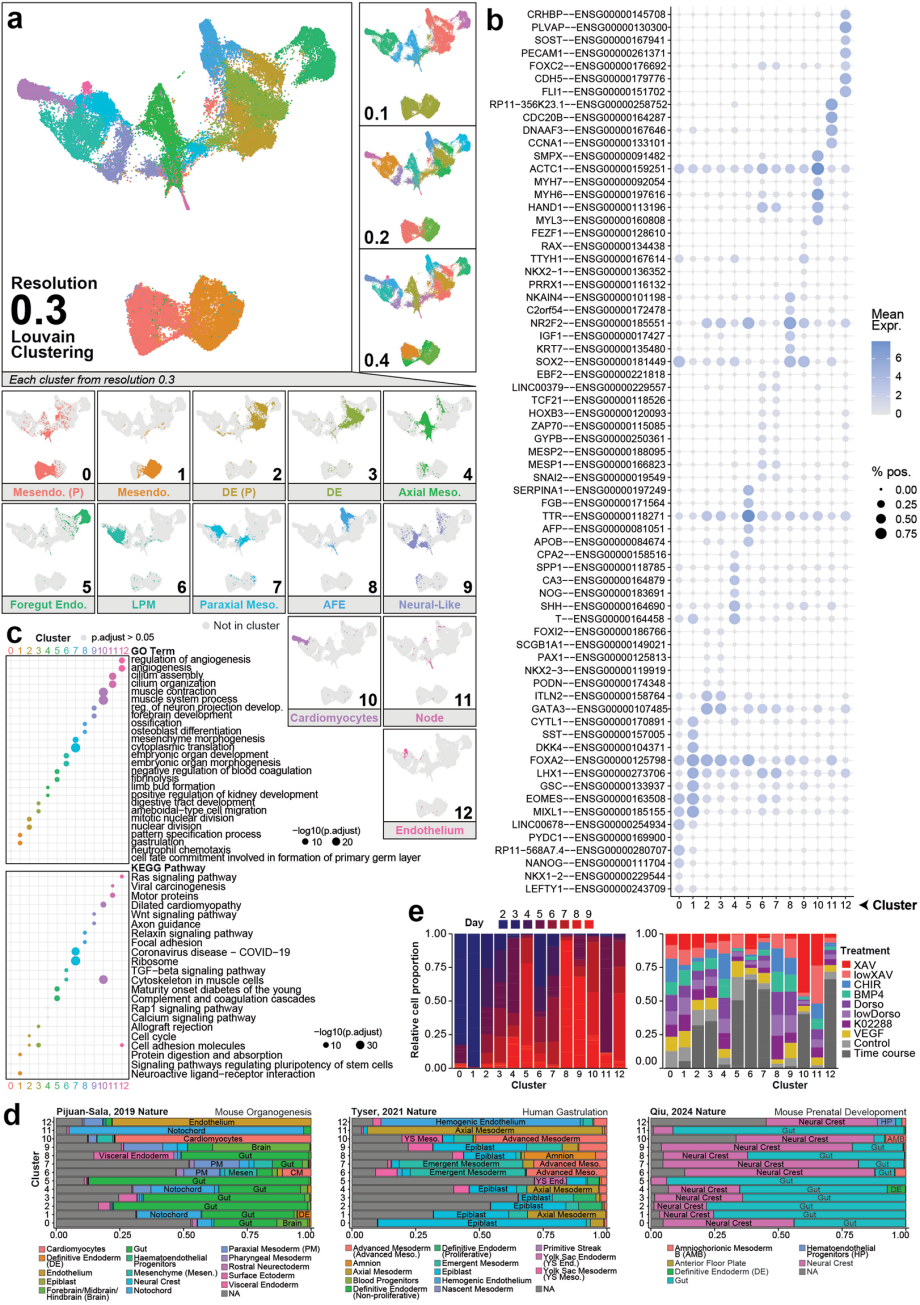
Cell type clustering and gene marker analysis reveal cell type heterogeneity in integrated dataset. (**a**) Louvain clustering of integrated data across four clustering resolutions (top). Resolution 0.3 is used for subsequent characterisation and each individual cluster is shown with cell type annotation (bottom). Mesendo.: Mesendoderm; (P): Proliferative; DE: Definitive Endoderm; Meso: Mesoderm; Endo.: Endoderm; LPM: Lateral Plate Mesoderm; AFE: Anterior Foregut Endoderm. (**b**) Mean expression and percentage of cells expressing select marker genes in each cluster. Marker gene list is a combination of selected marker genes based on prior knowledge and the top differentially expressed (DE) genes in each cluster. Full DE gene list for each cluster is provided in Supplementary Table S1. (**c**) Enrichment of GO term (top) and KEGG pathway (bottom) annotations based on the top 100 most significantly differentially expressed genes in each cluster. Shown are the top 2 highest ranking GO term or KEGG pathway for each cluster based on their Benjamini-Hochberg corrected p-value (p.adjust), where grey points have a corrected p-value above 0.05. The full list of enrichment results is provided in Supplementary Table S1. Reg. of neuron projection develop.: Regulation of neuron projection development. (**d**) Label transfer results evaluating the atlas dataset against three *in vivo* datasets of early development^6,19,20^. Bar plots show proportion of cells in each cluster annotated with each annotation. NA indicates cells with prediction scores below the chosen threshold (see **Methods**). (**e**) Proportion contribution of cells from each time point (left) and treatment condition (right) to each cell type cluster. Relative cell proportion for time point (left) is the normalised by total number of cells captured at each time point while relative cell proportion for treatment group (right) is normalised by the total number of cells captured at each time point and treatment group.

We performed cell clustering using the standard *Seurat* pipeline on four resolutions: 0.1, 0.2, 0.3, and 0.4 to characterise broad cell type heterogeneity in the integrated dataset. We selected the 0.3 resolution for further analysis of cell type heterogeneity in the integrated dataset (Fig. 4a). Differential gene expression analysis for each cluster, followed by GO term and KEGG pathway enrichment of the significantly differentially expressed genes in each of the 12 clusters provide a basis for cluster annotation (Fig. 4b,c & Supplementary Table S1) and enable evaluation of cell type assessments based on gene expression in Fig. 3c. We additionally used label transfer analysis in the *Seurat* pipeline to evaluate the clusters with reference to three scRNA-seq datasets of early development *in vivo* (Fig. 4d)^6,19,20^. Together, these data confirm that the unique cell types introduced in the signalling perturbation dataset to be axial mesoderm (Cluster 4: *TBXT*, *NOG*), anterior foregut endoderm (Cluster 8: *NR2F2*, *SOX2*), nodal cells (Cluster 11: *NOG*), and neural-like cells (Cluster 9: *SOX2*, *NR2F2*). Finally, we compute relative proportions of cells captured from each time point and treatment condition in each cluster to facilitate interpretation of how the signalling cues contribute to different cell fate outcomes (Fig. 4e). This confirms that the two clusters primarily at day 2 (Clusters 0 & 1) show roughly even contributions of cells from each treatment group, as anticipated since they were all untreated at the day 2 time point (see Fig. 1a). As another point of validation, Fig. 4e also shows that the cardiomyocyte cluster (Cluster 10) is strongly biased in the XAV treatment, aligning with expectations as treatment with this WNT inhibitor at the germ layer stage of differentiation is commonly used in cardiomyocyte differentiation protocols^26^. These two examples confirm recapitulation of expected treatment effects, providing confidence in the dataset’s utility for interpreting the impact of the less well-characterised signalling perturbations on lineage outcomes.

Taken together, this study provides a controlled scRNA-seq dataset capturing cell type dynamics resulting from signalling modulation of hPSC mesendoderm differentiation. By profiling over 60,000 cells across sequential stages of differentiation and perturbations of WNT, BMP, and VEGF signalling pathways, we offer a valuable reference point for benchmarking *in vitro* differentiation against *in vivo* development. The dataset enables dissection of dose-dependent signalling effects on lineage specification, offering insight into how pathway modulation influences fate decisions relevant to embryogenesis and organogenesis. This resource will thus support optimisation of high-fidelity cardiomyocyte differentiation protocols with utility across regenerative medicine, drug screening applications, and potential to inform synthetic biology and customised cell differentiation strategies.

## Supplementary information

Supplementary Table 1

## Data Availability

Code for the described data processing and analysis are provided at https://github.com/palpant-comp/data_descriptor.
